# 4493 New module supporting Community Engaged Research added to COALESCE (teamscience.net) online training for interdisciplinary research teams

**DOI:** 10.1017/cts.2020.218

**Published:** 2020-07-29

**Authors:** Bonnie Spring, Megha Patel, Angela F Pfammatter

**Affiliations:** 1 Northwestern University; 2 Northwestern University Feinberg School of Medicine

## Abstract

OBJECTIVES/GOALS: A new researcher-facing module to support community engaged research has been added to the updated COALESCE website and user traffic was tracked since last reporting. We describe the process of development and the features of the new module, past 2-year traffic, and plans to develop a community facing module. METHODS/STUDY POPULATION: We monitored the number of unique users of COALESCE (teamscience.net) between 2017 and 2019 to determine if traffic slowed, stayed the same, or increased since teamscience.net was physically updated to function on mobile devices. In December 2019, a new module was launched to introduce researchers to the stages of team science community engaged research. To develop the module, we collaborated with academic partners at University of Illinois-Chicago to identify 3 local historic research case studies in and to characterize how each exemplified a team science stage: assembly, launch, or maturation. After interviewing key team members from each study, we iterated storyboards and scripts in collaboration with community engaged research experts and case study team members. The module was built, tested, and launched. RESULTS/ANTICIPATED RESULTS: In the 6 years between 2011 through 2017, the site attracted 16,016 unique visitors (approximately 2699 per year). In 2 years from 2017 through 2019, since the modernization of the website, user traffic has held steady or grown, attracting 6992 unique visitors (approximately 3496 per year). Our newly posted researcher-facing module highlights team assembly in the case a task force charged with reducing disparity in breast cancer outcomes in Chicago, team launch in a study to improve asthma management in a local FQHC, and team maturation in a study comparing clinic-based to public school-based treatment of disruptive behavior. We will soon create a companion community-facing module and resources to address identified needs for community partners engaging in research with academic institutions. DISCUSSION/SIGNIFICANCE OF IMPACT: COALESCE (teamscience.net) remains the first and only open-access, online training in team science for health professionals. Recent updates have improved usability and expanded available resources. We launched a comprehensive module for academics interested in community engaged research; future work will develop parallel community facing resources.

